# Executive functions in children with developmental language disorder: a systematic review and meta-analysis

**DOI:** 10.3389/fnins.2024.1390987

**Published:** 2024-08-12

**Authors:** Tengfei Niu, Shiqiang Wang, Jingxi Ma, Xiaoping Zeng, Ruiling Xue

**Affiliations:** ^1^Department of Basic Courses, Chongqing Medical and Pharmaceutical College, Chongqing, China; ^2^Department of Neuro-Oncology, Chongqing University Cancer Hospital, Chongqing, China; ^3^Chongqing Medical University, Chongqing, China; ^4^Department of Neurology, Chongqing General Hospital, Chongqing, China; ^5^Department of Rehabilitation, Chongqing General Hospital, Chongqing, China

**Keywords:** developmental language disorder, executive functions, children, systematic review, meta-analysis

## Abstract

**Background:**

The purpose of the current study was to assess the differences between children with developmental language disorder (DLD) and typically developing (TD) children in their performance of executive functions from working memory, inhibitory control, and cognitive flexibility.

**Methods:**

We performed a systematical search of PubMed, Embase, Cochrane, and Web of Science for case control studies (published in English between January 1, 1950, and October 11, 2023) comparing the differences in the performance of executive functions between DLD and TD children.

**Results:**

Forty eligible studies were included in the present study (*N* = 3,168 participants). In comparison with TD children, DLD children exhibited significantly poorer performances in all six verbal working memory tasks (backward digit recall task, SMD –1.4321, 95% CI –2.2692 to –0.5950; listening recall task, SMD –1.4469, 95% CI –1.7737 to –1.1202; counting recall task, SMD –0.9192, 95% CI –1.4089 to –0.4295; digit recall task, SMD –1.2321, 95% CI –1.4397 to –1.0244; word list recall task, SMD –1.1375, 95% CI –1.5579 to –0.7171; non-word recall task, SMD –1.5355, 95% CI –1.8122 to –1.2589). However, regarding inhibitory control and cognitive flexibility, the differences between DLD and TD children depended on specific circumstances. In subgroup analyses of all verbal working memory tasks, DLD children presented notably lower performance than TD children in both the monolingual English and monolingual non-English groups, and in both the preschooler and school-aged groups.

**Conclusion:**

This study proves that verbal working memory deficits can be seen as a marker for children with DLD and are not affected by age or language type.

**Systematic Review Registration:**

https://www.crd.york.ac.uk/PROSPERO/display_record.php?RecordID=391070, CRD42023391070.

## 1 Introduction

Executive function (EF) is a comprehensive term that encompasses various intricate cognitive abilities. These abilities work together to regulate lesser cognitive skills, aiming to achieve a specific objective or goal (Doebel, 2020). Currently, EFs are most accurately depicted as a sophisticated collection of cognitive abilities, incorporating elements like working memory, inhibitory control, cognitive flexibility, planning, reasoning, as well as problem-solving (Jay, 2002). Classically, EFs have been subdivided into three domains: working memory, inhibition, and cognitive flexibility (Doebel, 2020). Language serves as an organized system relying on speech, gesture, signs, and writing. It allows us to communicate, express emotions, preferences, and thoughts, contemplate our surroundings, define our identity, and articulate our history and culture (Kamkar and Morton, 2017). The close connection between EF and language becomes evident, considering that language primarily functions through symbolic representation. This involves applying a set of rules to interpret symbols (grammar), arranging symbols in a specific order for meaning (syntax), and utilizing symbols in spoken language with adjustments in pitch, intensity, and emotion (prosody) (Purves et al., 2018).

A substantial body of research indicates a positive correlation between EF and various language skills. For example, studies demonstrate a connection between EF and vocabulary knowledge (Carlson et al., 2005), sentence reading (Lewis et al., 2006), reading comprehension (Booth and Boyle, 2009; Sesma et al., 2009; Booth et al., 2010; Butterfuss and Kendeou, 2018), and syntax (White et al., 2017). As for the relationship between the three components of EF and language, evidence indicates that working memory (WM) plays a role in children’s perception of complex syntax (Georgiou and Theodorou, 2023), language acquisition is influenced by the abilities of inhibitory control (Gandolfi and Viterbori, 2020; Usai et al., 2020; Yuile and Sabbagh, 2021), and children who possess superior cognitive flexibility may be more adept at applying the diverse rules of language (Blair and Raver, 2015). Conversely, studies have shown that language could directly influence the development of EFs (Doebel, 2020; Merchán et al., 2022). Some theories propose that language skills come into play during the execution of executive tasks (Winsler et al., 2009). Furthermore, Zelazo and colleagues proposed that language serves as a foundational precursor to the emergence of EF in children (Zelazo et al., 2003). Empirical findings from longitudinal studies have provided further support for the notion that early language abilities can predict subsequent EF skills (Marcovitch and Zelazo, 2009; Vallotton and Ayoub, 2011; Kuhn et al., 2016).

Developmental language disorder (DLD), which is previously known as specific language impairment (SLI), is a condition that is characterized by difficulties in learning and utilizing language without any accompanying neurological damage, hearing loss, or cognitive impairment, as well as a lack of behavioral or emotional disorders (Bishop et al., 2017). As a condition that can affect various aspects of language such as phonology, morphology, lexicon, syntax, and pragmatics, DLD persists from preschool to adolescence and adulthood and can lead to challenges in social, emotional, academic, and vocational domain (Bishop, 2006; Brizzolara et al., 2011; St Clair et al., 2011). Therefore, examining EFs in DLD children could be especially significant in correctly understanding these children’s language and communication challenges.

According to recent studies, children with DLD may exhibit poorer performance in various oral language skills and EFs when compared to their typically developing (TD) peers (Henry et al., 2012; Leonard, 2014; Lukács et al., 2016). In exploring EFs, both cognitive tasks and behavioral evaluations are employed. Cognitive tasks have been utilized to study various critical components of EFs in children with DLD. However, there is significant variability in the number of studies that examine different components.

Working memory (WM) is the most studied key component of EFs among children with DLD. Most of these studies find evidence that children with DLD demonstrate poorer performance when compared with TD, age-matched children on WM tasks that require the storage and processing of auditory information (Dodwell and Bavin, 2008; Coady et al., 2010; Duinmeijer et al., 2012; Claessen et al., 2013; Engel de Abreu et al., 2014; Delage and Durrleman, 2018; Evans et al., 2018; Arslan et al., 2020; Balilah and Archibald, 2022). However, whether DLD children perform worse in visuospatial WM compared with TD peers is inconclusive (Henry et al., 2012; Engel de Abreu et al., 2014; Lukács et al., 2016; Balilah and Archibald, 2022), although Vugs et al. discovered significant visuospatial WM deficits in DLD children in a meta-analysis (Vugs et al., 2013; Kapa et al., 2017).

In terms of inhibitory control, Pauls et al. identified a significant difference between DLD and TD children in a meta-analysis (Pauls and Archibald, 2016). However, a study using the Bielefeld Screening for Early Recognition of Dyslexia (BISC) test found no significant differences between the DLD and TD children (Reichenbach et al., 2016). What’s more, Agnes Lukacs et al. reported controversial inhibitory control results in DLD children (Lukács et al., 2016).

Regarding cognitive flexibility, like the inhibitory control, Pauls et al. identified a significant difference between DLD and TD children in a meta-analysis (Pauls and Archibald, 2016). However, both the study using the task-switching test (Lukács et al., 2016) and the study using the Dimensional Change Card Sort (DCCS) test (Reichenbach et al., 2016) found no significant differences between the DLD and TD children.

Although the above studies on verbal WM have shown relatively consistent results, little attention has been paid to the effects of age and language on EFs in children with DLD; and studies on nonverbal WM inhibitory control, and cognitive flexibility have shown mixed results. Moreover, in previous meta-analyses (Vugs et al., 2013; Pauls and Archibald, 2016; Kapa et al., 2017) subtle discrepancies among different outcome measures were concealed by amalgamating an extensive array of data from various measurement tasks. Hence, we conducted this systematic review and meta-analysis to address the relationship between EFs and DLD children from an evidence-based perspective. In this study, to ensure the comparability of data, when an outcome is analyzed, we only pooled the data measured by the same experimental task or the same outcome indicator. For the most studied WM, we adopted Baddeley’s multicomponent WM model, comparing the performance of DLD and TD children in terms of each specific subcomponent (Baddeley, 2003). Besides, based on subgroup analyses, we further explored whether preschoolers and school-aged children presented significant differences, and whether monolingual English-speaking and monolingual non-English-speaking children had significant differences in terms of performance on WM. What’s more, this meta-analysis included not only neurocognitive measures but also the rating scale of behavior BRIEF-P in assessing different components of EFs to check whether the result depended on the specific measurement method. In addition, the current study is, to the best of our knowledge, the first systematic review and meta-analysis investigating all three key components of EFs when comparing differences in EF performance between DLD and TD children. Based on the above, the following research questions are proposed:

1. Compared with TD children, do DLD children perform significantly worse on Baddeley’s four WM subcomponents, verbal inhibitory control and nonverbal inhibitory control, and verbal cognitive flexibility and nonverbal cognitive flexibility after controlling task types?
2. Is the performance of DLD children on the three major components of EFs affected by age and language compared to TD children?
3. Do the neurocognitive measures and the rating scale of behavior BRIEF-P get the same results in assessing different components of EFs?

## 2 Materials and methods

This systematic review and meta-analysis was reported in accordance with the Preferred Reporting Items for Systematic Reviews and Meta-Analyses (PRISMA 2020) Statement and was registered at International Prospective Register of Systematic Reviews (No.: CRD42023391070).

### 2.1 Selection criteria

#### 2.1.1 Inclusion criteria

1. In the included studies, in comparison with the TD children, children with DLD were diagnosed by a speech-language pathologist, with test scores indicating at least 1.25 SDs below the mean on various language tests (Tomblin et al., 1996).
2. There was no remarkable difference between DLD and TD children in their performance on nonverbal intelligence test (Bishop, 2014).
3. To ensure the comparability of data, for WM, we only included studies in which the outcome was measured by the same experimental task, including Backward Digit Recall, Counting Recall, Listening Recall, Working Memory Test Battery for Children, Wechsler Intelligence Scale for Children, Automated Working Memory Assessment, Backward Block Tapping, Odd-one-out, Spatial Span, Digit Recall, Nonword Recall, Word List Recall, Block Recall, Dot Matrix.

#### 2.1.2 Exclusion criteria

Duplicates; case reports, abstracts, conference papers, reviews, meta-analyses, and other non-original articles; full text not available or original research data not extractable; studies in which none of the three outcomes (WM, inhibition, and cognitive flexibility) were reported; studies in which Intelligence Quotient (IQ) of participants were not controlled; studies in which the WM task was different from that of all the other included studies; and studies from which the data obtained was not comparable to those of other included studies.

### 2.2 Search strategy

We collected relevant studies that were published between January 1, 1950, and Dec 31, 2022, by a systematic search of Embase, PubMed, Cochrane, and Web of Science. The language was limited to English. To reduce the risk of missing newly published literature, we conducted a supplementary search on October 11, 2023. The detailed search strategy can be seen in Supplementary Table S1.

### 2.3 Study selection and data extraction

We imported the retrieved literature into EndNote, excluded duplicates, read the titles and abstracts to screen the original studies for initial matches and downloaded the full text, and then finalized the included literature by reading the full text. Before carrying out data extraction, we developed a data extraction spreadsheet containing first author, publication year, study design type, language used by the participants, total number of participants, age (mean [SD]), sex, outcomes reported. Literature screening and data extraction were completed independently by two researchers and cross-checked upon completion, with a third researcher assisting in the adjudication of disputes if needed.

### 2.4 Quality evaluation

Two authors independently finished quality evaluation of the eligible studies using the Newcastle-Ottawa Scale (NOS), which included eight items and was divided into three dimensions: selection, comparability, and exposure (Wells et al., 2014). Quality scores on the NOS had a range from 0 to 9, with studies scoring 7 or more being rated as having high quality, those scoring 4 to 6 as having medium quality, and those scoring 3 or less as having low quality.

### 2.5 Outcomes

Working memory refers to a system with limited capacity responsible for temporarily maintaining and storing information in an active, online state during cognitive tasks (Baddeley, 2003). The multicomponent WM model developed by Baddeley has been widely utilized in research concerning children with DLD (Baddeley and Hitch, 1974; Baddeley, 2003). This model posits a central executive (CE) system connected to subsystems such as the phonological loop, the visuospatial sketchpad, as well as the episodic buffer. The CE system is responsible for coordinating and controlling activities within WM, but its limited attentional capacity necessitates attentional control. Since the phonological loop and visuospatial sketchpad are in charge of temporarily storing verbal and visuospatial information, respectively, they are referred to as “slave” systems. The episodic buffer, a more recent addition to the model, is believed to facilitate the integration of information from various sources into meaningful chunks (Baddeley, 2003). Because working memory is the most studied outcome and the one that was further subdivided into four components, in this meta-analysis, based on Baddeley’s model, WM is divided into four components: verbal CE, visuospatial CE, phonological loop, and visuospatial storage. Only studies that measured a subcomponent of working memory with the same experimental task were eligible to be included in this meta-analysis. Of the included literature, the verbal CE contained three tasks Backward Digit Recall, Counting Recall, and Listening Recall; the visuospatial CE included three tasks Backward Block Tapping, Odd-one-out, and Spatial Span; the phonological loop covered three tasks Digit Recall, Nonword Recall, and Word List Recall; and the visuospatial storage contained Block Recall, Dot Matrix, and Mazes Memory. Given that a task can be tested differently, this study also conducted subgroup analyses based on the specific test method.

According to Diamond’s definition, inhibition, also known as inhibitory control, involves avoiding being distracted by external factors or refraining from making a predominant but erroneous response (Diamond, 2013). To ensure comparability of data, in this study, verbal inhibition was analyzed according to the measurement of accuracy; nonverbal inhibition was analyzed according to the measurement of accuracy, reaction time, and error numbers, respectively.

Cognitive flexibility, also known as shifting or task switching, is the capacity to modify one’s thoughts and actions in response to changes in the environment (Seiferth and Thienel, 2013). In this study, cognitive flexibility was statistically analyzed separately by verbal and non-verbal cognitive flexibility.

### 2.6 Statistical analysis

We assessed the difference between DLD and TD children on three outcomes: WM, inhibitory control, and cognitive flexibility.

The pooled estimates of the mean differences between DLD and TD children were calculated using a random-effects model or a fixed-effects model. The results were analyzed by calculating the SMD and the 95% CI. We used the Cochran *I*^2^ test to assess heterogeneity across the studies, with *I*^2^ > 50% suggesting moderate-to-high heterogeneity, and *I*^2^ less than or equal to 50% as low heterogeneity. When *I*^2^ was greater than 50%, a random-effects model was used for meta-analysis and when I^2^ was less than or equal to 50%, a fixed-effects model was adopted. Here’s a simplified explanation of how the Cochran *I*^2^ test works (Huedo-Medina et al., 2006):

1. *Q* Test: The *I*^2^ statistic is derived from the *Q* statistic, which is calculated as part of the meta-analysis process. The *Q* statistic follows a chi-square distribution and tests the null hypothesis that all studies are estimating the same effect size.
2. Degrees of Freedom (df): The degrees of freedom for the *Q* test are calculated as the number of studies minus one (k – 1).
3. *I*^2^ Calculation: The *I*^2^ statistic is then calculated using the formula:

*I*^2^ $=\left(\frac{Q-df}{Q}\right)$ × 100%, where *Q* is the Cochran’s *Q* heterogeneity statistic.

For the three outcomes, we also analyzed subgroups according to the specific test method, age (the preschooler and the school-aged subgroups), and language (English and non-English subgroups). We used R (version 4.3.1) for meta-analysis.

## 3 Results

### 3.1 Study selection

**Figure 1:**
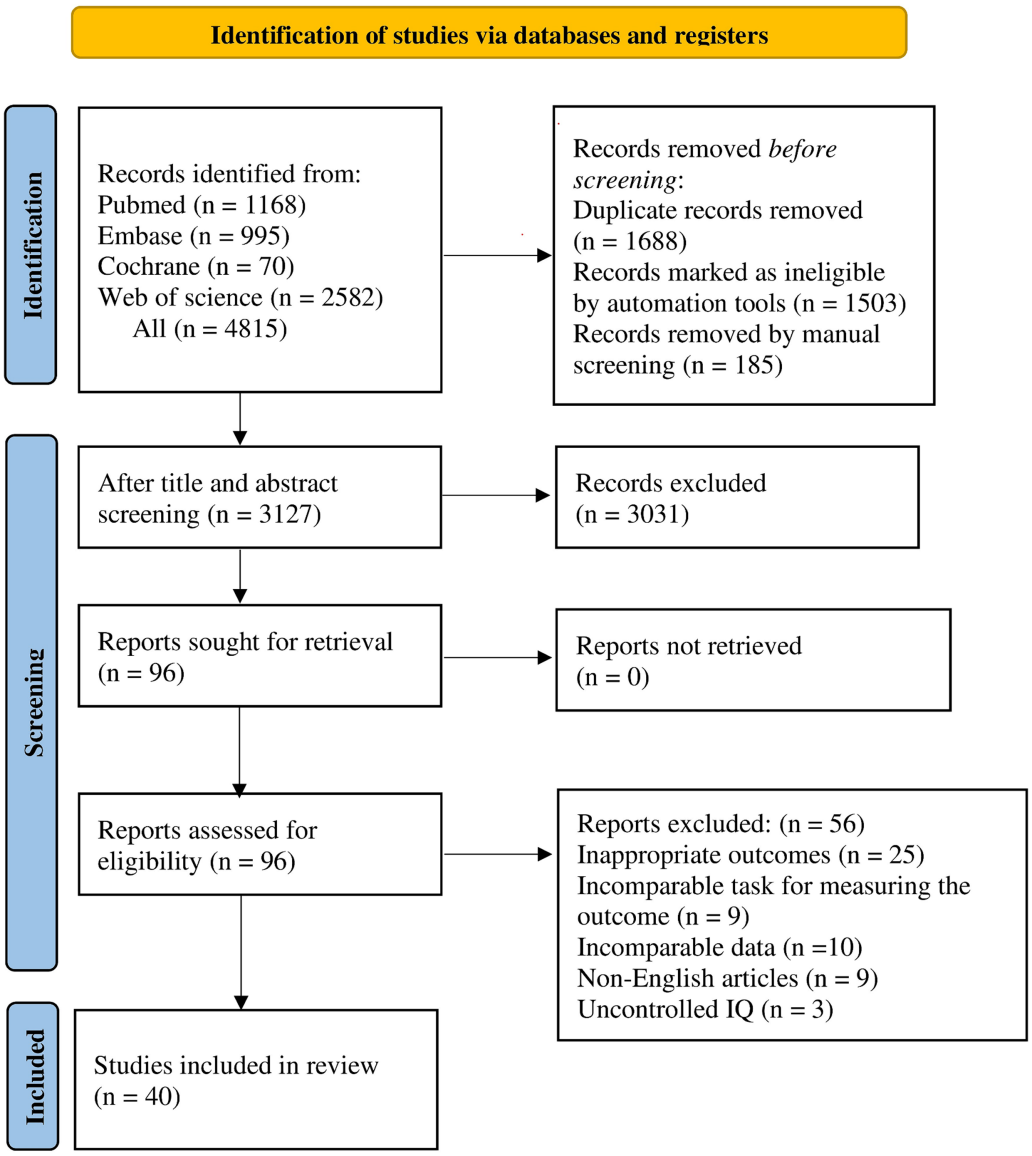
Flow chart of literature screening.

We initially retrieved 4,815 reports, and after excluding 1,688 duplicate publications, we read the titles and abstracts of the remaining 3,127 reports and screened 96 studies relevant to our topic. After reading the full text of 96 reports, we found that 25 reports did not report the outcome relevant to this meta-analysis, the tasks for measuring the outcomes in 9 reports could not be compared with those of the included studies, and the data for the outcomes in 10 reports could not be compared with the data of the included studies, 9 reports were non-English, and 3 reports did not control the IQ of the children studied. After further exclusion of the 56 reports mentioned above, a final total of 40 studies (Dodwell and Bavin, 2008; Coady et al., 2010; Lum and Zarafa, 2010; Mainela-Arnold et al., 2010; Spanoudis and Natsopoulos, 2011; Duinmeijer et al., 2012; Farrant et al., 2012; Henry et al., 2012; Hutchinson et al., 2012; Lum et al., 2012; Lum and Bleses, 2012; Claessen et al., 2013; Wittke et al., 2013; Engel de Abreu et al., 2014; Taylor et al., 2014; Vugs et al., 2014, 2017; Jackson et al., 2016; Lukács et al., 2016, 2017; Reichenbach et al., 2016; Kapa et al., 2017; Kuusisto et al., 2017; Laloi et al., 2017; Delage and Durrleman, 2018; Evans et al., 2018; Schaeffer et al., 2018; Fyfe et al., 2019; Ladányi and Lukács, 2019; Sikora et al., 2019; Arslan et al., 2020; Guiberson and Rodríguez, 2020; Kapa and Erikson, 2020; Marini et al., 2020; Gillam et al., 2021; Ralli et al., 2021; Balilah and Archibald, 2022; Kalliontzi et al., 2022; Thomas et al., 2022) were included (Figure 1).

### 3.2 Study characteristics and quality assessment of the included studies

The 40 studies included in this meta-analysis were all case–control studies containing 1,307 children with DLD (age range 3.44–11.53 years) and 1861 TD children (age range 3.52–10.7 years). Thirty-four papers reported gender matched DLD children and TD children, while the remaining 6 papers did not disclose the gender of the participants. Of all 40 studies, 37 studies reported on working memory; 11 studies reported on inhibitory control, and 7 studies reported on cognitive flexibility. In terms of language, 18 studies dealt with monolingual English-speaking participants, 18 studies focused on monolingual non-English-speaking participants, 4 studies reported bilingual participants, and 2 studies had both monolingual and bilingual participants. In terms of age, 15 studies reported preschoolers and 28 studies reported school-aged children. Based on the NOS scores, 7 studies were of moderate quality, while the other 33 were of high quality (Table 1).

**Table 1:** Characteristics of eligible studies.

**First Author**	**Year**	**Language**	**Sample Size** **(DLD/TD)**	**Mean Age (Standard Deviation) (DLD/TD)**	**Male/Female (DLD; TD)**	**Outcomes Reported**	**NOS**
Eleni Kalliontzi	2022	Monolingual, Greek	53/62	4.45 (0.2)/4.47 (0.18)	36/17; 26/36	Nonverbal inhibition, Verbal and nonverbal cognitive flexibility	8
Areej M. A. Balilah	2022	Monolingual, Greek	52/369	8.33 (1.00)/7.92 (1.12)	19/33;139/230	Working memory (verbal phonological loop, verbal CE, visuospatial storage, visuospatial CE)	6
Sheila Thomas	2022	Monolingual, English	30/30	3.44 (0.26)/3.52 (0.24)	19/11; 15/15	Working memory (verbal phonological loop), Nonverbal inhibition	6
		Bilingual	30/30	3.45 (0.27)/3.52 (0.24)	18/12; 15/15		
Asimina M. Ralli	2021	Monolingual, Greek	29/29	8.6/8.9	15/14; 12/17	Working memory (verbal CE), Nonverbal inhibition	8
Ronald B. Gillam	2021	Monolingual, English	117/117	9.5/9.5	67/50; 74/43	Working memory (verbal phonological loop), Verbal cognitive flexibility	8
Leah L. Kapa	2020	Mostly monolingual, English with 16 bilingual children	41/41	4.95 (0.44)/4.97 (0.45)	15/26; 15/26	Working memory (verbal phonological loop, verbal CE), Verbal inhibition, Nonverbal cognitive flexibility	7
Andrea Marini	2020	Monolingual, Italian	16/24	5.19 (0.03)/5.43 (0.46)	12/4; 15/9	Working memory (verbal phonological loop, verbal CE)	9
Emily Jackson	2020	Monolingual, English	50/54	6.96 (0.64)/6.84 (0.63)	36/14;30/24	Working memory (verbal phonological loop, verbal CE, visuospatial storage)	7
MarkM. Guibeson	2019	Bilingual	37/93	4.6 (0.77)/4.6 (0.77)	18/19; 44/49	Working memory (verbal phonological loop)	8
Katarzyna Sikora	2019	Monolingual, Dutch	33/41	10.1/10.7	Not Available	Working memory (visuospatial CE), Nonverbal inhibition	7
Seckin Arslan	2019	Monolingual, French	12/24	8.94/8.85	10/2; 20/4	Working memory (verbal phonological loop, verbal CE, visuospatial storage, visuospatial CE)	8
Julia L. Evans	2018	Monolingual, English	60/87	9.63 (1.23)/9.66 (1.37)	Not Available	Working memory (verbal phonological loop), Verbal cognitive flexibility, Verbal inhibition	7
Emily R. Fyfe	2018	Monolingual, English	18/18	10.4 (1.8)/10.0 (1.9)	10/8; 6/12	Working memory (verbal CE)	6
Helene Delage	2018	Mixed (French monolingual+ bilingual)	21/21	9.83 (2.83)/age-matched	14/7;14/7	Working memory (verbal phonological loop, verbal CE)	6
Enikő Ladányi	2018	Monolingual, Hungarian	31/31	8.92 (1.17)/8.92 (1.08)	Matched on gender	Working memory (verbal CE)	7
Jeannette Schaeffer	2017	Monolingual, Dutch	27/27	9.58 (2.17)/9.83 (2.17)	Matched on gender	Working memory (verbal phonological loop, verbal CE, visuospatial CE)	9
Leah L.Kapa	2017	Monolingual, English	26/26	4.93 (0.49)/4.96 (0.4)	13/13; 13/13	Working memory (visuospatial CE), Verbal inhibition, Verbal and nonverbal cognitive flexibility	7
Aude Laloi	2017	Monolingual, French	19/17	7.5 (0.7)/7.3 (0.1)	10/9;10/7	Nonverbal inhibition	7
		Bilingual	13/19	8.0 (0.7)/7.4 (0.7)	8/5;7/12		
Brigitte Vugs	2017	Monolingual, Dutch	30/33	4.76 (0.60)/4.92 (0.58)	21/9; 20/13	Working memory (verbal phonological loop, verbal CE, visuospatial storage, visuospatial CE)	7
		Monolingual, Dutch	30/33	7.99 (0.50)/8.35 (0.61)	21/9; 20/13	Working memory (verbal phonological loop, verbal CE, visuospatial storage, visuospatial CE)	
Agnes Kukacs	2017	Monolingual, Hungarian	21/21	8.89 (1.06)/8.85 (1.03)	15/6;15/6	Working memory (verbal phonological loop)	8
Marika A. Kuusisto	2016	Monolingual, Finnish	22/22	8.2 (0.6)/8.3 (0.7)	4/18; 4/18	Working memory (BRIEF), Inhibition (BRIEF), Cognitive flexibility (BRIEF)	7
Agnes Lukacs	2016	Monolingual, Hungarian	31/31	7.84 (1.81)/7.81 (1.81)	23/8; 23/8	Working memory (verbal phonological loop, verbal CE, visuospatial storage, visuospatial CE), Verbal inhibition	7
Katrin Reichenbach	2016	Monolingual, German	30/30	5.28 (0.36)/5.18 (0.31)	22/8; 13/17	Working memory (verbal phonological loop), Verbal inhibition, Nonverbal cognitive flexibility	6
Emily Jackson	2016	Monolingual, English	23/26	5.37 (0.34)/5.49 (0.25)	18/5;10/16	Working memory (verbal phonological loop)	7
Brigitte Vugs	2014	Monolingual, Dutch	58/58	4.09 (0.62)/4.11 (0.57)	42/16; 32/26	Working memory (verbal phonological loop, verbal CE, visuospatial storage, visuospatial CE, BRIEF), Inhibition (BRIEF), Cognitive flexibility (BRIEF)	8
Engel de Abreu	2014	Bilingual, Portuguese/Luxembourgish	15/33	8.0 (0.62)/8.17 (0.08)	13/2;15/18	Working memory (verbal phonological loop, verbal CE, visuospatial storage, visuospatial CE), Nonverbal inhibition	7
Mary Claessen	2013	Monolingual, English	21/21	7.59 (0.3)/7.58 (0.35)	Not Available	Working memory (verbal phonological loop, verbal CE)	6
Lauren J.Taylor	2013	Monolingual, English	19/61	8.22 (2.13)/ 8.86 (1.66)	15/4;33/28	Working memory (verbal phonological loop)	8
Lucy A. Henry	2012	Monolingual, English	41/88	11.53 (1.33)/9.83 (2.36)	28/13; 59/29	Working memory (verbal CE, visuospatial CE), Nonverbal inhibition	9
Iris Duinmeijer	2012	Monolingual, Dutch	34/38	7.4 (1.05)/7.9 (1.09)	25/9; 17/21	Working memory (verbal phonological loop)	7
Brad M. Farrant	2012	Monolingual, English	30/30	5.25 (0.75)/5.23 (0.7)	26/4;26/4	Nonverbal cognitive flexibility	9
Kacie Wittke	2011	Monolingual, English	19/19	4.11 (0.46)/4.13 (0.47)	12/7; 12/7	Inhibition (BRIEF)	7
Esther Hutchinson	2011	Monolingual, English	18/24	7.77 (0.21)/7.67 (0.14)	12/6; 12/12	Working memory (verbal phonological loop, verbal CE, visuospatial storage)	7
Jarrad A.G. Lum	2011	Monolingual, Danish	13/20	7.71 (0.84)/7.92 (0.72)	Not Available	Working memory (verbal phonological loop, verbal CE)	6
Jarrad A.G. Lum	2011	Monolingual, English	51/51	9.8 (0.74)/9.85 (0.71)	35/16; 35/16	Working memory (verbal phonological loop, verbal CE, visuospatial storage)	8
George C. Spanoudis	2011	Monolingual, Greek	50/50	10.21 (0.86)/9.93 (1.03)	33/17; 39/11	Working memory (verbal phonological loop)	8
Jarrad A. G. Lum	2010	Monolingual, English	16/16	10.17 (0.89)/9.75 (0.72)	12/4; 12/4	Working memory (verbal phonological loop, verbal CE)	7
Elina Mainela	2010	Monolingual, English	16/16	10 (0.97)/10.10 (1.14)	Not Available	Working memory (verbal phonological loop)	7
Jeffry Coady	2010	Monolingual, English	18/18	9.17/8.83	8/10;6/12	Working memory (verbal phonological loop)	7
Kristy Dodwell	2008	Monolingual, English	16/25	6.7 (0.25)/6.7 (0.35)	Not Available	Working memory (verbal phonological loop), Nonverbal inhibition	7

### 3.3 Meta-analysis

#### 3.3.1 Working memory (WM)

##### 3.3.1.1 WM-verbal CE

###### 3.3.1.1.1 Merging of effect sizes

Thirteen studies reported on the Backward Digit Recall (BDR) task, and a meta-analysis with a random-effects model (*I*^2^ = 88.5%) revealed that children in the TD group significantly outperformed children in the DLD group (SMD = –1.4321, 95% CI: −2.2692 to –0.5950). Five studies reported the Counting Recall (CR) task, and a meta-analysis with a random-effects model (*I*^2^ = 86.6%) revealed that children in the DLD group underperformed significantly compared with those in the TD group (SMD = –0.9192, 95% CI: −1.4089 to –0.4295). Eleven studies reported on the Listening Recall (LR) task, and a meta-analysis using a random-effects model (*I*^2^ = 76.5%) demonstrated that children in the TD group performed remarkably better compared with those in the DLD group (SMD = –1.4469, 95% CI: −1.7737 to –1.1202) (Supplementary Table S2).

###### 3.3.1.1.2 Subgroup analyses

In our subgroup analyses by the specific test, the BDR task was dominated by the Working Memory Test Battery for Children (WMTB-C, studies = 4) and the Wechsler Intelligence Scale for Children (WIS-C, studies = 5); the CR task included the Automated Working Memory Assessment (AWMA, studies = 3) and WMTB-C (Studies = 2); and the WMTB-C (Studies = 6) predominated in the LR task. For the different tests of the BDR, CR and LR tasks, children in the DLD group performed significantly worse than children in the TD group (Supplementary Table S2). For age subgroups, in both the preschooler subgroup and the school-aged subgroup the TD children significantly outperformed their respective DLD children in the three tasks of BDR, CR, and LR (*p* < 0.05). Similarly, for the language subgroups, in both the English and non-English subgroups the TD children significantly outperformed their respective DLD children (*p* < 0.05) in the above three tasks (Supplementary Table S2).

##### 3.3.1.2 WM-visuospatial CE

###### 3.3.1.2.1 Merging of effect sizes

Two studies reported Backward Block Tapping (BBT) task, and a fixed-effects model (*I*^2^ = 0.0%) was adopted for meta-analysis revealing that children in the TD group significantly outperformed children in the DLD group (SMD = –0.6089, 95% CI: −1.0469 to –0.1709). Odd-one-out (OOO) task was reported in seven studies, and a random-effects model (*I*^2^ = 85.8%) was used for the meta-analysis, which suggested that children in the DLD group performed significantly worse than children in the TD group (SMD = -0.4923, 95% CI: −0.9140 to –0.0706). Four studies reported Spatial Span (SS) task, and the meta-analysis was completed with a random-effects model (*I*^2^ = 77.7%), showing that children in the TD group significantly outperformed children in the DLD group (SMD = –0.6787, 95%CI: −1.0812 to –0.2762) (Supplementary Table S3).

###### 3.3.1.2.2 Subgroup analyses

In our subgroup analysis by the specific test, OOO task was mainly implemented through Odd-one-out Test (Henry, 2001, studies = 3) and Automated Working Memory Assessment (Alloway 2007, studies = 3). According to the Odd-one-out Test (Henry, 2001), the DLD group performed remarkably worse compared with the TD group (*p* < 0.05), but AWMA revealed no significant difference between the DLD group and the TD group (*p* > 0.05) (Supplementary Table S3).

For both OOO and SS tasks, in the subgroup of preschoolers, the DLD children performed remarkably worse than the TD counterparts (*p* < 0.05), but no significant difference was observed between the DLD and the TD children in the school-age subgroup (*p* > 0.05). Based on the subgroup analysis by language in OOO task, significant differences were found between the DLD and the TD children in the English subgroup (*p* < 0.05), while no significant difference was observed between the DLD and the TD children in the non-English subgroup (*p* > 0.05) (Supplementary Table S3).

##### 3.3.1.3 WM-phonological loop

###### 3.3.1.3.1 Merging of effect sizes

A random-effects model (*I*^2^ = 64.7%) was employed for the meta-analysis of Digit Recall (DR) task in 18 studies, and the results showed that children in the DLD group remarkably underperformed children in the TD group (SMD = –1.2321, 95% CI: −1.4397 to –1.0244). Nineteen studies reported on the Nonword Recall (NWR) task, and the meta-analysis using a random-effects model (*I*^2^ = 79.1%) indicated that children in the TD group significantly outperformed children in the DLD group (SMD = –1.5355, 95% CI: –1.8122 to –1.2589). Seven studies reported the Word List Recall (WLR) task and a random-effects model (*I*^2^ = 82.0%) was adopted for meta-analysis. The result revealed that children in the DLD group had significantly worse performance than children in the TD group (SMD = –1.1375, 95% CI: −1.5579 to –0.7171) (Supplementary Table S4).

###### 3.3.1.3.2 Subgroup analyses

In our subgroup analyses by the specific test, the DR task was dominated by WMTB-C (Studies = 3), WIS-C (Studies = 4), and AWMA (Studies = 5); the NWR task mainly included the NWR Task (Dollaghan and Campbell 1998, studies = 7), and the WLR was dominated by the AWMA (Studies = 1) and WMTB-C (Studies = 2). Children in the DLD group remarkably underperformed children in the TD group in all tests of the DR, NWR, and WLR tasks (Supplementary Table S4).

In our subgroup analyses by age, in both the preschooler subgroup and the school-aged subgroup the DLD children performed significantly worse (*p* < 0.05) compared to their respective TD children in the DR, NWR, and WLR tasks. In the subgroup analyses by language, DLD children in both the English and non-English subgroups performed significantly worse (*p* < 0.05) compared to their respective TD children in the DR, NWR and WLR tasks (Supplementary Table S4).

##### 3.3.1.4 WM-visuospatial CE

###### 3.3.1.4.1 Merging of effect sizes

Seven studies reported the Block Recall (BR) task, and a random-effects model (*I*^2^ = 65.8%) was employed for meta-analysis, which revealed that children in the TD group performed significantly better than children in the DLD group (SMD = –0.3569, 95% CI: −0.6361 to –0.0777). Five studies reported the Dot Matrix (DM) task, and a random-effects model (*I*^2^ = 85.4%) was employed for meta-analysis, which showed that children in the DLD group markedly underperformed children in the TD group (SMD = –0.6561, 95% CI: −1.1879 to –0.1244). Three studies reported the Mazes Memory (MM) task, and a meta-analysis with a random effects model (*I*^2^ = 91.7%) indicated no significant difference between the DLD and TD groups (SMD = –0.5525, 95% CI: −1.4187 to 0.3137) (Supplementary Table S5).

###### 3.3.1.4.2 Subgroup analyses

In our subgroup analysis by the specific test, BR task mainly included WMTB-C (Studies = 3) and AWMA (Studies = 2). WMTB-C test showed that the DLD group markedly underperformed the TD group, while other test methods showed no significant difference between the DLD group and the TD group. Unlike the condition in BR task, WMTB-C (Studies = 2) in MM task showed no significant difference between the DLD group and the TD group.

For all the BR, DM, and MM tasks, in the preschooler subgroup the DLD children markedly underperformed TD children (*p* < 0.05), whereas no significant difference was observed between the DLD and the TD children in the school-aged subgroup (*p* > 0.05). Language subgroup analysis in BR task showed that in the English subgroup, the DLD children significantly performed worse than the TD children (*p* < 0.05), while in the non-English subgroup, the difference between the two groups was not significant (*p* > 0.05). Contrary to the above result, language subgroup analysis in MM task showed no significant difference between DLD and TD children in the English subgroup (*p* > 0.05), but significant differences were observed between DLD and TD children in the non-English subgroup (*p* < 0.05).

#### 3.3.2 Inhibitory control

##### 3.3.2.1 Merging of effect sizes

Three studies reported verbal inhibition with Accuracy Rate as the outcome indicator, and the meta-analysis using a random-effects model (*I*^2^ = 97.7%) indicated that the difference between the DLD and TD groups was not significant (SMD = -1.9915, 95% CI: −4.5188 to 0.5358). Three studies reported nonverbal inhibition with Accuracy Rate as the outcome indicator, and the meta-analysis using a random-effects model (*I*^2^ = 94.6%) revealed that the DLD children markedly underperformed the TD counterparts (SMD = –1.9343, 95% CI: −3.4606 to –0.4079). Another six studies reported nonverbal inhibition using Reaction Time as the outcome indicator, and the meta-analysis using a random-effects model (*I*^2^ = 94.5%) demonstrated that the difference between the DLD and TD children was not significant (SMD = 1.1651, 95% CI: −0.2643 to 2.5944). Two other studies reported nonverbal inhibition using Error Numbers as the outcome indicator, and the meta-analysis using a random-effects model (*I*^2^ = 57.2%) revealed that the TD children markedly outperformed the DLD children (SMD = 0.6643, 95% CI: 0.1092 to 1.2193) (Supplementary Table S6).

##### 3.3.2.2 Subgroup analyses

In the experiments where nonverbal inhibition was measured using Reaction Time as the outcome indicator, no significant difference was found between the DLD children and their respective TD children in either the preschooler subgroup or the school-aged subgroup (*p* > 0.05), as well as between the DLD and TD children in the non-English subgroup (*p* > 0.05), whereas TD children performed better compared to DLD children in the bilingual subgroup (Supplementary Table S6).

#### 3.3.3 Cognitive flexibility

##### 3.3.3.1 Merging of effect sizes

Four included studies measured verbal cognitive flexibility and the meta-analysis with a fixed-effects model (*I*^2^ = 0.0%) suggested a marked worse performance in children with DLD than in children with TD (SMD = –0.4690, 95% CI: −0.6397 to –0.2982). Nonverbal cognitive flexibility was measured in five studies and the meta-analysis using a random-effects model (*I*^2^ = 96.9%) revealed that the difference between DLD and TD children was not significant (SMD –1.9792, 95% CI: –4.7906 to 0.8323) (Supplementary Table S7).

##### 3.3.3.2 Subgroup analyses

In experiments measuring verbal cognitive flexibility, in both the preschool and school-age subgroups DLD children performed significantly worse (*p* < 0.05) compared to their respective TD children, and in both the English and non-English subgroups the DLD children performed significantly worse (*p* < 0.05) compared to their respective TD children. In the experiment measuring nonverbal cognitive flexibility, no significant difference was observed between the DLD and TD children in both the English subgroup and the non-English subgroup (Supplementary Table S7).

#### 3.3.4 BRIEF-parent behavioral measurement

Three studies tested elements of EF using the BRIEF-parent scale, and the meta-analysis with a fixed-effects model (*I*^2^ = 0.0%) revealed that the DLD children performed significantly worse compared to TD children (SMD = 0.837, 95% CI: 0.657 to 1.018). Subgroup analyses of the three main components WM, Inhibition, and Cognitive Flexibility indicated that children with DLD markedly underperformed TD children on all three components (*p* < 0.05) (Supplementary Table S8).

## 4 Discussion

Our findings from 40 studies involving 3,168 participants demonstrate that in neurocognitive tasks DLD children exhibited notably lower performance than TD children in verbal WM. However, regarding visuospatial WM, whether the difference between DLD and TD children is significant depends on the specific task. In terms of verbal inhibitory control, no significant difference was found between the DLD and TD children whereas as for nonverbal inhibitory control, whether there is a significant difference between DLD and TD children depends on how the outcome was measured. With respect to verbal cognitive flexibility, children with DLD markedly underperformed TD children, whereas there was no significant difference between children with DLD and TD children in terms of nonverbal cognitive flexibility. In contrast, across all measured outcomes using the BRIEF-P Scale, DLD children presented markedly worse performance compared with TD children.

A few recent studies have also revealed that children with DLD had significantly poorer performance than TD children on verbal WM tasks (Guiberson and Rodríguez, 2020; Kapa and Erikson, 2020; Jackson et al., 2021; Larson and Ellis Weismer, 2022). Consistent with these studies, the present analysis provides further support and elaboration on previous discoveries in several aspects. Unlike previous studies, the current analysis further divided verbal WM into two components, verbal CE and verbal storage, and measured them separately using three tasks, yielding the same result as previous studies. Furthermore, since Klara Marton et al. found that the performance of WM is influenced by the linguistic attributes of the language accessed, the present analysis further divided children into monolingual English and monolingual non-English subgroups (Marton et al., 2007). It concluded that children with DLD underperformed TD children on verbal WM in all languages. We believe that although each language has its specific linguistic attributes that can affect verbal WM performance, the effects of these linguistic attributes on the WM performance in children with DLD and TD children are similar under a particular language and so do not affect the result that children with DLD perform significantly worse than TD children on verbal WM tasks. Given that single studies can only focus on a particular age group and that children at different ages have different levels of neurological development, the present analysis further divided the children into preschool and school-aged subgroups and concluded that children with DLD underperformed TD children on verbal WM in both preschool and school-aged subgroups.

In a prior meta-analysis, Vugs et al. found deficits in both storage and CE components of visuospatial WM in children with DLD (Vugs et al., 2013). Consistent with Vugs’s study, DLD children had significant weaker CE capacities on three visuospatial WM tasks than TD children in this study. However, regarding visuospatial storage, two of three tasks revealed that the difference between DLD and TD children was significant, while another MM task showed that the difference between the two groups was not significant. We believe that the different results between this analysis and Vugs’s study are mainly because Vugs’s data comes from different tasks. By combining a wide range of data collected from various measurement tasks, the subtle discrepancies among different measurement tasks in Vugs’s study were masked. Unlike Vugs’s analysis, in this study, only data obtained by the same experimental task were included and compared to ensure the accuracy of the analysis. Given that MM task was only reported in three papers, more original studies are needed in the future to explore further results.

Through subgroup analysis, we found that in the preschool subgroup children with DLD markedly underperformed TD children in all visuospatial WM tasks, but that in the school-aged subgroup children with DLD did not differ significantly from TD children in all the tasks. The above findings can be interpreted in two ways. One possibility is that at preschool age, the elements of EF have not yet undergone complete differentiation so other EF elements may influence visuospatial WM performance on the specific task. As EF elements differentiate in late childhood, children’s performance on visuospatial WM tasks can reflect their actual level. It is the gradual emergence of distinct EF elements that contribute to different performances between the preschool group and the school-aged group. Several studies, including Shing et al. and Gandolfi et al. have discovered evidence supporting this gradual emergence of EF elements (Shing et al., 2010; Gandolfi et al., 2014). The other possible reason is that the potential difficulties in verbal and visuospatial skills during preschool years might decrease as children with DLD develop visual recoding abilities over time, allowing them to compensate for their verbal impairments while maintaining their visual capacity (Engel de Abreu et al., 2014). What’s more, the subgroup analysis based on the age factor demonstrated that in the English subgroup the DLD children performed significantly worse than the TD children in both the visuospatial CE and storage tasks. In contrast, in the non-English subgroup, the DLD children did not differ significantly from its TD counterparts. We believe that specific characteristics of different languages may affect DLD children’s visuospatial WM abilities, depending on the language they speak.

In terms of inhibitory control, Archibald and Leonard found that although children with DLD underperformed TD children, this difference was insignificant (Archibald and Gathercole, 2006; Leonard, 2014). In this study, the meta-analysis on inhibitory control is partially consistent with the above result. Given that the analysis only included a limited number of studies, more original studies are needed in the future to explore further results. And another meta-analysis by Pauls et al. concluded that children with DLD were significantly worse on inhibitory control and cognitive flexibility than TD children (Pauls and Archibald, 2016). In this study, we further divided cognitive flexibility into the verbal part and the nonverbal part and got a partially different conclusion. We believe that this difference may be due to two factors. One is that Pauls’s study did not present the original data. Because of the different data conversion standards, the accuracy of the data included in the study may have been affected, which in turn may have further affected the result of the data analysis. Second, his study was a comparative analysis of data obtained through different experimental tasks, ignoring the differences in the different experimental tasks themselves.

In addition to the findings based on the neurocognitive tasks described above, the BRIEF-P scale was used to assess the performance in children with DLD and TD children in everyday life. This behavioral measure revealed that children with DLD markedly underperformed TD children on the three main components of EF. This above finding is consistent with that in the study of Cuperus et al. (2014). We believe that the different results between neurocognitive tasks and the behavioral scale BRIEF-P is because in neurocognitive tasks we can influence the results by artificially adjusting the difficulty of the task, whereas the problems children encounter in their everyday life are more natural and flexible.

## 5 Limitations

There are several limitations of the current meta-analysis. The strict inclusion criteria for this study, whereby the included comparable experimental data were derived from the same task, objectively limited the amount of included literature. Of the 21 sets of data analysis in this study, 9 sets of data analysis included less than 5 pieces of literature, so the results of this study must be treated with caution. Furthermore, heterogeneity was high in 16 of the 22 sets of data analysis, particularly in all three neurocognitive tasks of the three outcomes, making it even more essential for us to treat the results of this study with caution. The author suggests that the high heterogeneity of these data analyses may be due to the differences in task difficulty and the way scores were tallied across experiments, even though each set of data originated from the same experimental task. What’s more, this study does not divide DLD children into receptive and productive DLD subtypes to compare their performance in EFs.

## 6 Conclusion and clinical implications

In conclusion, this research reinforces the idea that deficits in verbal WM can be considered a marker for children with DLD. The extent to which children with DLD differ from TD children in visuospatial WM performance varies depending on the specific neurocognitive task. The performance of children with DLD on visuospatial WM tasks remains unaffected by language and age, whereas age influences their performance on visuospatial WM tasks. Moreover, different languages may influence the performance of children with DLD on visuospatial WM tasks. Further studies are needed to explore the difference in the performance of children with DLD on inhibitory control and cognitive flexibility.

This study has important clinical implications. First, it suggests that professionals should consider multiple aspects of EF such as WM, inhibitory control, and cognitive flexibility when assessing and treating children with DLD. Second, it supports the development and implementation of targeted interventions to improve EF in children with DLD, potentially improving their language ability and other related cognitive skills. Third, the study suggests that when we design interventions, we should take into account that children with DLD may have different needs and responses at different ages and stages of development. Moreover, by designing a measure encompassing both neurocognitive and behavioral modalities, we will comprehensively assess the EFs of children with DLD to formulate interventions, allowing for further development of the children’s EFs.

## Data availability statement

The original contributions presented in the study are included in the article/Supplementary material, further inquiries can be directed to the corresponding authors.

## Author contributions

TN: Conceptualization, Supervision, Writing – original draft, Writing – review & editing. SW: Formal analysis, Investigation, Methodology, Writing – original draft. JM: Formal analysis, Investigation, Methodology, Writing – original draft. XZ: Resources, Supervision, Writing – original draft. RX: Formal analysis, Funding acquisition, Writing – review & editing.
