# Supplementary material for: Executive functions in children with developmental language disorder: a systematic review and meta-analysis
Source: Front Neurosci. 2024 Aug 12;18:1390987. doi: 10.3389/fnins.2024.1390987 (PMC11345193; doi:10.3389/fnins.2024.1390987)
Supplement: Supplementary file 1 [file Table_1.docx]

# Supplementary Information

**Table S1** Search strategy

**Table S2** Verbal central executive

**Table S3** Visuospatial central executive

**Table S4** Phonological loop

**Table S5** Visuospatial storage

**Table S6** Inhibition

**Table S7** Cognitive flexibility

**Table S8** BRIEF

# Table S1 Search strategy

**1.Pubmed**

| **Search number** | **Query** | **Results** |
| --- | --- | --- |
| #1 | "Language Development Disorders"[Mesh] | 7,366 |
| #2 | (((((((((((Language Development Disorders[Title/Abstract]) OR (Language Development Disorder[Title/Abstract])) OR (Developmental Language Disorders[Title/Abstract])) OR (Developmental Language Disorder[Title/Abstract])) OR (Speech Delay[Title/Abstract])) OR (Speech Delays[Title/Abstract])) OR (Semantic-Pragmatic Disorder[Title/Abstract])) OR (Semantic Pragmatic Disorder[Title/Abstract])) OR (Semantic-Pragmatic Disorders[Title/Abstract])) OR (Central Auditory Processing Disorder[Title/Abstract])) OR (Language Delay[Title/Abstract])) OR (Language Delays[Title/Abstract]) | 4,153 |
| #3 | #1 OR #2 | 9,845 |
| #4 | Specific Language Disorder[MeSH Terms] | 71 |
| #5 | (((Specific Language Disorder[Title/Abstract]) OR (Specific Language Disorders[Title/Abstract])) OR (Specific Language Impairment[Title/Abstract])) OR (Specific Language Impairments[Title/Abstract]) | 1,888 |
| #6 | #4 OR #5 | 1,895 |
| #7 | #3 OR #6 | 10,715 |
| #8 | Child[MeSH Terms] | 2,159,689 |
| #9 | ((Child[Title/Abstract]) OR (children[Title/Abstract])) OR (Preschoolers[Title/Abstract]) | 1,516,455 |
| #10 | #8 OR #9 | 2,627,895 |
| #11 | Typically developing[Title/Abstract] | 14,002 |
| #12 | #7 AND #10 AND #11 | 1,168 |

**2.Cochrane**

| **Search number** | **Query** | **Results** |
| --- | --- | --- |
| #1 | MeSH descriptor: [Language Development Disorders] explode all trees | 258 |
| #2 | (Language Development Disorders):ti,ab,kw OR (Language Development Disorder):ti,ab,kw OR (Developmental Language Disorders):ti,ab,kw OR (Developmental Language Disorder):ti,ab,kw OR (Speech Delay):ti,ab,kw | 1828 |
| #3 | (Speech Delays):ti,ab,kw OR (Semantic-Pragmatic Disorder):ti,ab,kw OR (Semantic Pragmatic Disorder):ti,ab,kw OR (Semantic-Pragmatic Disorders):ti,ab,kw OR (Central Auditory Processing Disorder):ti,ab,kw | 172 |
| #4 | (Language Delay):ti,ab,kw OR (Language Delays):ti,ab,kw | 883 |
| #5 | #1 OR #2 OR #3 OR #4 | 2515 |
| #6 | MeSH descriptor: [Specific Language Disorder] explode all trees | 3 |
| #7 | (Specific Language Disorder):ti,ab,kw OR (Specific Language Disorders):ti,ab,kw OR (Specific Language Impairment):ti,ab,kw OR (Specific Language Impairments):ti,ab,kw | 1415 |
| #8 | #6 OR #7 | 1415 |
| #9 | #5 OR #8 | 3506 |
| #10 | MeSH descriptor: [Child] explode all trees | 78477 |
| #11 | (Child):ti,ab,kw OR (children):ti,ab,kw OR (Preschoolers):ti,ab,kw | 179187 |
| #12 | #10 OR #11 | 179187 |
| #13 | (Typically developing):ti,ab,kw | 1049 |
| #14 | #9 AND #12 AND #13 | 70 |

**3.Embase**

| **Search number** | **Query** | **Results** |
| --- | --- | --- |
| #1 | 'developmental language disorder'/exp | 7402 |
| #2 | 'language development disorders':ab,ti OR 'language development disorder':ab,ti OR 'developmental language disorders':ab,ti OR 'developmental language disorder':ab,ti OR 'speech delay':ab,ti OR 'speech delays':ab,ti OR 'semantic-pragmatic disorder':ab,ti OR 'semantic pragmatic disorder':ab,ti OR 'semantic-pragmatic disorders':ab,ti OR 'central auditory processing disorder':ab,ti OR 'language delay':ab,ti OR 'language delays':ab,ti | 5393 |
| #3 | 'specific language disorder':ab,ti OR 'specific language disorders':ab,ti OR 'specific language impairment':ab,ti OR 'specific language impairments':ab,ti | 2029 |
| #4 | #1 OR #2 OR #3 | 11586 |
| #5 | 'child'/exp | 3380368 |
| #6 | child:ab,ti OR children:ab,ti OR preschoolers:ab,ti | 1960887 |
| #7 | #5 OR #6 | 3841889 |
| #8 | 'typically developing':ab,ti | 17032 |
| #9 | #4 AND #7 AND #8 | 995 |

**4.Web of science**

| **Search number** | **Query** | **Results** |
| --- | --- | --- |
| #1 | "Language Development Disorders (Topic) OR Language Development Disorder (Topic) OR Developmental Language Disorders (Topic) OR Developmental Language Disorder (Topic) OR Speech Delay (Topic) OR Speech Delays (Topic) OR Semantic-Pragmatic Disorder (Topic) OR Semantic Pragmatic Disorder (Topic) OR Semantic-Pragmatic Disorders (Topic) OR Central Auditory Processing Disorder (Topic) OR Language Delay (Topic) OR Language Delays (Topic) OR Specific Language Disorder (Topic) OR Specific Language Disorders (Topic) OR Specific Language Impairment (Topic) OR Specific Language Impairments (Topic) | 42668 |
| #2 | "Child (Topic) OR children (Topic) OR Preschoolers (Topic) | 1994133 |
| #3 | "Typically developing (Topic) | 103422 |
| #4 | "#3 AND #2 AND #1 | 2582 |

| **Table S2 Verbal central executive** | | | | | | |
| --- | --- | --- | --- | --- | --- | --- |
| **No** | **Task** | **Subgroup** | **Studies** | **I2(%)** | **Model** | **SMD (95%CI)** |
| 1 | backward digit recall | **Test method** |  |  |  |  |
|  |  | Working Memory Test Battery for Children (Pickering and Gathercole, 2001) | 4 | 0.0 | Fixed | -1.0246[-1.2641; -0.7851] |
|  |  | Automated Working Memory Assessment (Alloway, 2007) | 2 | 84.3 | Random | -0.6710[-1.2815; -0.0605] |
|  |  | Wechsler Intelligence Scale for Children (Wechsler, 2003) | 5 | 0.0 | Fixed | -1.2989[-1.6082; -0.9896] |
|  |  | Test not specified | 1 | NA | Fixed | -7.2083[-8.6087; -5.8079] |
|  |  | CELF-IV | 1 | NA | Fixed | -0.2761[-0.8841; 0.3320] |
|  |  | **Age** |  |  |  |  |
|  |  | preschoolers | 3 | 0.0 | Fixed | -1.0568 [-1.3179; -0.7957] |
|  |  | school-age children | 10 | 91.3 | Random | -1.5742 [-2.7137; -0.4347] |
|  |  | **Language** |  |  |  |  |
|  |  | English | 4 | 47.5 | Fixed | -0.9209 [-1.1652; -0.6766] |
|  |  | Non-English | 9 | 91.9 | Random | -1.7227 [-2.9686; -0.4768] |
|  |  | **Overall** | 13 | 88.5 | Random | -1.4321 [-2.2692; -0.5950] |
| 2 | counting recall | **Test method** |  |  |  |  |
|  |  | Automated Working Memory Assessment (Alloway, 2007) | 3 | 92.0 | Random | -0.8391[-1.6415; -0.0368] |
|  |  | Working Memory Test Battery for Children (Pickering and Gathercole, 2001) | 2 | 26.2 | Fixed | -1.1164[-1.4706; -0.7623] |
|  |  | **Age** |  |  |  |  |
|  |  | preschoolers | 1 | NA | Fixed | -1.6250 [-2.0465; -1.2035] |
|  |  | school-age children | 4 | 76.8 | Random | -0.7264 [-1.1691; -0.2838] |
|  |  | **Language** |  |  |  |  |
|  |  | English | 2 | 26.2 | Fixed | -1.1164 [-1.4706; -0.7623] |
|  |  | Non-English | 3 | 92.0 | Random | -0.8391 [-1.6415; -0.0368] |
|  |  | **Overall** | 5 | 86.6 | Random | -0.9192 [-1.4089; -0.4295] |
| 3 | listening recall | **Test method** |  |  |  |  |
|  |  | Working Memory Test Battery for Children (Pickering and Gathercole, 2001) | 6 | 71.2 | Random | -1.2525[-1.6749; -0.8300] |
|  |  | Automated Working Memory Assessment (Alloway, 2007) | 3 | 28.7 | Fixed | -1.9812[-2.2923; -1.6701] |
|  |  | Competing Language Processing Task (Gaulin & Campbell, 1994) | 2 | 0 | Fixed | -1.1915[-1.6772; -0.7057] |
|  |  | **Age** |  |  |  |  |
|  |  | preschoolers | 2 | 0 | Fixed | -2.1517 [-2.5229; -1.7805] |
|  |  | school-age children | 9 | 60.1 | Random | -1.2578 [-1.5529; -0.9627] |
|  |  | **Language** |  |  |  |  |
|  |  | English | 6 | 69.8 | Random | -1.2509 [-1.6863; -0.8156] |
|  |  | Non-English | 5 | 69 | Random | -1.6602 [-2.0922; -1.2282] |
|  |  | **Overall** | 11 | 76.5 | Random | -1.4469 [-1.7737; -1.1202] |

| **Table S3 Visuospatial central executive** | | | | | | |
| --- | --- | --- | --- | --- | --- | --- |
| **No** | **Task** | **Subgroup** | **Studies** | **I^2^(%)** | **Model** | **SMD (95%CI)** |
| 1 | backward block tapping | **Overall** | 2 | 0.0 | Fixed | -0.6089 [-1.0469; -0.1709] |
| 2 | odd-one-out | **Test method** |  |  |  |  |
|  |  | Odd-one-out Test (Henry,2001) | 3 | 28.3 | Fixed | -0.6303[-0.8972; -0.3634] |
|  |  | Automated Working Memory Assessment (Alloway, 2007) | 3 | 92 | Random | -0.1521[-0.9672; 0.6631] |
|  |  | Odd-one-out Test (Conway et al. 2005) | 1 | NA | Fixed | -1.0368[-1.5262; -0.5475] |
|  |  | **Age** |  |  |  |  |
|  |  | preschoolers | 1 | NA | Fixed | -0.9616 [-1.3466; -0.5765] |
|  |  | school-age children | 6 | 84.8 | Random | -0.4089 [-0.8731; 0.0552] |
|  |  | **Language** |  |  |  |  |
|  |  | English | 1 | NA | Fixed | -0.6081 [-0.9863; -0.2300] |
|  |  | Non-English | 6 | 87.8 | Random | -0.4721 [-0.9738; 0.0296] |
|  |  | **Overall** | 7 | 85.8 | Random | -0.4923 [-0.9140; -0.0706] |
| 3 | spatial span | **Age** |  |  |  |  |
|  |  | preschoolers | 2 | 0.0 | Fixed | -0.9900 [-1.3013; -0.6787] |
|  |  | school-age children | 2 | 56.8 | Random | -0.3736 [-0.8081; 0.0610] |
|  |  | **Overall** | 4 | 77.7 | Random | -0.6787 [-1.0812; -0.2762] |

| **Table S4 Phonological loop** | | | | | | |
| --- | --- | --- | --- | --- | --- | --- |
| **No** | **Task** | **Subgroup** | **Studies** | **I^2^(%)** | **Model** | **SMD (95%CI)** |
| 1 | digit recall | **Test method** |  |  |  |  |
|  |  | Wechsler Intelligence Scale for Children (Wechsler, 2003) | 4 | 17.4 | Fixed | -1.2984[-1.6375; -0.9592] |
|  |  | Automated Working Memory Assessment (Alloway, 2007) | 5 | 87.9 | Random | -1.3063[-1.8903; -0.7223] |
|  |  | Working Memory Test Battery for Children (Pickering and Gathercole, 2001) | 3 | 0.0 | Fixed | -1.0489[-1.3157; -0.7820] |
|  |  | Clinical Evaluation of Language Functioning – IV (Semel et al., 2003a) | 1 | NA | Fixed | -1.1711[-1.8304; -0.5118] |
|  |  | Comprehensive Test of Phonological Processing (Wagner, Torgesen, & Rashotte, 1999) | 1 | NA | Fixed | -1.0567[-1.8024; -0.3110] |
|  |  | Kaufman Assessment Battery for Children (2003) | 1 | NA | Fixed | -1.0718[-1.7448; -0.3988] |
|  |  | Not specified | 3 | 79.2 | Random | -1.4458[-2.1592; -0.7325] |
|  |  | **Age** |  |  |  |  |
|  |  | preschoolers | 5 | 76.3 | Random | -1.3841 [-1.8923; -0.8760] |
|  |  | school-age children | 13 | 52.1 | Random | -1.1487 [-1.3622; -0.9353] |
|  |  | **Language** |  |  |  |  |
|  |  | English | 7 | 0.0 | Fixed | -1.0084 [-1.1797; -0.8372] |
|  |  | Non-English | 11 | 77.3 | Random | -1.3769 [-1.7046; -1.0492] |
|  |  | **Overall** | 18 | 64.7 | Random | -1.2321 [-1.4397; -1.0244] |
| 2 | nonword recall | **Test method** |  |  |  |  |
|  |  | Non-word Repetition Task (Dollaghan and Campbell,1998) | 7 | 82.6 | Random | -1.5251[-2.0470; -1.0032] |
|  |  | Non-word Repetition Task (Rispens& Baker, 2012) | 1 | NA | Fixed | -2.8223[-3.5897; -2.0548] |
|  |  | Arabic Nonword Repetition task (Shaalan, 2010) | 1 | NA | Fixed | -0.7154[-1.0098; -0.4211] |
|  |  | Automated Working Memory Assessment (Alloway, 2007) | 1 | NA | Fixed | -1.5682[-1.9860; -1.1504] |
|  |  | Working Memory Test Battery for Children (Pickering and Gathercole, 2001) | 1 | NA | Fixed | -1.1660[-1.5870; -0.7450] |
|  |  | Nonword Repetition(Gathercole & Baddeley, 1996) | 1 | NA | Fixed | -1.5435[-2.2457; -0.8413] |
|  |  | Developmental Neuropsychological Assessment-II (NEPSY-II; Korkman et al.2007) | 1 | NA | Fixed | -1.4893[-2.0559; -0.9227] |
|  |  | Nonword Repetition Task (Lukacs A et al., 2012) | 1 | NA | Fixed | -2.7592[-3.6241; -1.8943] |
|  |  | Non-word Repetition Task ((Ebert et al., 2008) | 1 | NA | Fixed | -1.1974[-1.6060; -0.7888] |
|  |  | Comprehensive Test of Phonological Processing (Wagner, Torgesen, & Rashotte, 1999) | 1 | NA | Fixed | -1.8715[-2.7190; -1.0240] |
|  |  | Not specified | 1 | NA | Fixed | -1.3751[-1.8126; -0.9377] |
|  |  | Nonword Repetition Test (Chiat and Polisenska, 2016) | 2 | 79.1 | Random | -1.4506[-2.3465; -0.5547] |
|  |  | **Age** |  |  |  |  |
|  |  | preschoolers | 6 | 78.9 | Random | -1.7378 [-2.2710; -1.2045] |
|  |  | school-age children | 13 | 76.1 | Random | -1.4373 [-1.7606; -1.1140] |
|  |  | **Language** |  |  |  |  |
|  |  | English | 12 | 73.6 | Random | -1.5227 [-1.8323; -1.2130] |
|  |  | Non-English | 5 | 90.7 | Random | -1.7850 [-2.5759; -0.9940] |
|  |  | Bilingual | 2 | 0.0 | Fixed | -1.1277 [-1.4533; -0.8020] |
|  |  | **Overall** | 19 | 79.1 | Random | -1.5355 [-1.8122; -1.2589] |
| 3 | word list recall | **Test method** |  |  |  |  |
|  |  | Automated Working Memory Assessment (Alloway, 2007) | 1 | NA | Fixed | -2.2268[-2.6930; -1.7607] |
|  |  | Working Memory Test Battery for Children (Pickering and Gathercole, 2001) | 2 | 0.0 | Fixed | -1.1795[-1.5358; -0.8231] |
|  |  | Dutch version of the California Verbal Learning Test, Children’s Version; Kalverboer and Deelman 1964) | 1 | NA | Fixed | -0.7501[-1.2296; -0.2706] |
|  |  | Not specified | 3 | 49.0 | Fixed | -0.7986[-1.0743; -0.5229] |
|  |  | **Age** |  |  |  |  |
|  |  | preschoolers | 3 | 86.1% | Random | -1.4570 [-2.2421; -0.6719] |
|  |  | school-age children | 4 | 52.6% | Random | -0.8790 [-1.2241; -0.5339] |
|  |  | **Language** |  |  |  |  |
|  |  | English | 4 | 0.0 | Fixed | -1.1255 [-1.3860; -0.8650] |
|  |  | Non-English | 3 | 93.9 | Random | -1.1590 [-2.2103; -0.1077] |
|  |  | **Overall** | 7 | 82 | Random | -1.1375 [-1.5579; -0.7171] |

| **Table S5 Visuospatial storage** | | | | | | |
| --- | --- | --- | --- | --- | --- | --- |
| **No** | **Task** | **Subgroup** | **Studies** | **I^2^(%)** | **Model** | **SMD (95%CI)** |
| 1 | block recall | **Test method** |  |  |  |  |
|  |  | Working Memory Test Battery for Children (Pickering and Gathercole, 2001) | 3 | 0.0 | Fixed | -0.3988[-0.6509; -0.1468] |
|  |  | Automated Working Memory Assessment (Alloway, 2007) | 2 | 92.8 | Random | -0.5685[-1.4728; 0.3358] |
|  |  | Corsi block tapping test (Corsi, 1972) | 1 | NA | Fixed | 0.0000[-0.6930; 0.6930] |
|  |  | Corsi span task (not specified) | 1 | NA | Fixed | -0.0316[-0.5294; 0.4663] |
|  |  | **Age** |  |  |  |  |
|  |  | preschoolers | 2 | 75.6 | Random | -0.7555 [-1.3125; -0.1985] |
|  |  | school-age children | 5 | 0.0 | Fixed | -0.1728 [-0.3648; 0.0191] |
|  |  | **Language** |  |  |  |  |
|  |  | English | 3 | 0.0 | Fixed | -0.3988 [-0.6509; -0.1468] |
|  |  | Non-English | 4 | 82.5 | Random | -0.3202 [-0.8264; 0.1860] |
|  |  | **Overall** | 7 | 65.8 | Random | -0.3569 [-0.6361; -0.0777] |
| 2 | dot matrix | **Age** |  |  |  |  |
|  |  | preschoolers | 2 | 0.0 | Fixed | -1.2660 [-1.5884; -0.9436] |
|  |  | school-age children | 3 | 53.4 | Random | -0.2834 [-0.6737; 0.1070] |
|  |  | **Overall** | 5 | 85.4 | Random | -0.6561 [-1.1879; -0.1244] |
| 3 | mazes memory | **Test method** |  |  |  |  |
|  |  | Automated Working Memory Assessment (Alloway, 2007) | 1 | NA | Fixed | -1.4119[-1.8201; -1.0038] |
|  |  | Working Memory Test Battery for Children (Pickering and Gathercole, 2001) | 2 | 0.0 | Fixed | -0.1018[-0.4297; 0.2260] |
|  |  | **Age** |  |  |  |  |
|  |  | preschoolers | 1 | NA | Fixed | -1.4119 [-1.8201; -1.0038] |
|  |  | school-age children | 2 | 0.0 | Fixed | -0.1018 [-0.4297; 0.2260] |
|  |  | **Overall** | 3 | 91.7 | Random | -0.5525 [-1.4187; 0.3137] |

| **Table S6 Inhibition** | | | | | | |
| --- | --- | --- | --- | --- | --- | --- |
| **No** | **Outcome and Measurement** | **Subgroup** | **Studies** | **I^2^(%)** | **Model** | **SMD (95%CI)** |
| 1 | verbal inhibition-accuracy | NA | 3 | 97.7 | Random | -1.9915 [-4.5188; 0.5358] |
| 2 | nonverbal inhibition-accuracy | NA | 3 | 94.6 | Random | -1.9343 [-3.4606; -0.4079] |
| 3 | nonverbal inhibition-reaction time | **Age** |  |  |  |  |
|  |  | preschoolers | 1 | NA | Fixed | 0.1084 [-0.2585; 0.4754] |
|  |  | school-age children | 5 | 94.9 | Random | 1.3862 [-0.3018; 3.0742] |
|  |  | **Language** |  |  |  |  |
|  |  | non-English | 4 | 96.7 | Random | 1.4198 [-0.8017; 3.6413] |
|  |  | bilingual | 2 | 28.2 | Fixed | 0.7134 [0.2359; 1.1909] |
|  |  | **Overall** | 6 | 94.5 | Random | 1.1651 [-0.2643; 2.5944] |
| 4 | nonverbal inhibition-error numbers | NA | 2 | 57.2 | Random | 0.6643 [0.1092; 1.2193] |

| **Table S7 Cognitive flexibility** | | | | | | |
| --- | --- | --- | --- | --- | --- | --- |
| **No** | **Outcome** | **Subgroup** | **Studies** | **I^2^(%)** | **Model** | **SMD (95%CI)** |
| 1 | verbal cognitive flexibility | **Age** |  |  |  |  |
|  |  | preschoolers | 2 | 0.0 | Fixed | -0.4517 [-0.7600; -0.1433] |
|  |  | school-age children | 2 | 0.0 | Fixed | -0.4766 [-0.6817; -0.2716] |
|  |  | **Language** |  |  |  |  |
|  |  | English | 3 | 0.0 | Fixed | -0.4480 [-0.6400; -0.2561] |
|  |  | non-English | 1 | NA | Fixed | -0.5484 [-0.9220; -0.1747] |
|  |  | **Overall** | 4 | 0.0 | Fixed | -0.4690 [-0.6397; -0.2982] |
| 2 | nonverbal cognitive flexibility | **Task** |  |  |  |  |
|  |  | DCCS (dimensional change card sort task) | 4 | 97.5 | Random | -2.4305[-5.9108; 1.0499] |
|  |  | Sorting card task (Yang, 2015) | 1 | NA | Fixed | -0.2299[-0.5978; 0.1380] |
|  |  | **Language** |  |  |  |  |
|  |  | English | 3 | 98.0 | Random | -3.2126 [-7.6786; 1.2533] |
|  |  | non-English | 2 | 0.0 | Fixed | -0.1972 [-0.4976; 0.1033] |
|  |  | **Overall** | 5 | 96.9 | Random | -1.9792 [-4.7906; 0.8323] |

| **Table S8 BRIEF** | | | | | | |
| --- | --- | --- | --- | --- | --- | --- |
| **No** | **Task** | **Subgroup** | **Studies** | **I^2^(%)** | **Model** | **SMD (95%CI)** |
| 1 | BRIEF-parent | working memory | 2 | 0.0 | Fixed | 0.9847 [0.6557; 1.3137] |
| 2 | BRIEF-parent | inhibition | 3 | 0.0 | Fixed | 0.6273 [0.3414; 0.9133] |
| 3 | BRIEF-parent | cognitive flexibility | 2 | 0.0 | Fixed | 0.9670 [0.6383; 1.2957] |
